# “Optimizing empathic self-disclosures in dignity therapy: Improving the patient–provider relationship for more humanistic palliative care”

**DOI:** 10.1017/S1478951525000306

**Published:** 2025-04-28

**Authors:** Mila Yunita, Nanda Alfan Kurniawan, Husni Hanafi, Palasara Brahmani Laras, Fiki Prayogi, Hendra Pribadi, Mint Husen Raya Aditama

**Affiliations:** 1 Guidance and Counseling, Universitas Negeri Surabaya, Surabaya, Indonesia; 2 Faculty of Education, Guidance and Counseling, Universitas Mulawarman, Samarinda, Indonesia; 3 Guidance and Counseling, Universitas Negeri Malang, Malang, Indonesia; 4 Guidance and Counseling, Universitas Mercu Buana Yogyakarta, Yogyakarta, Indonesia; 5 Guidance and Counselling, TKIP PGRI Bandar Lampung, Bandar Lampung, Indonesia; 6 Guidance and Counseling, Universitas Borneo Tarakan, Tarakan, Indonesia; 7 Guidance and Counseling, Universitas Negeri Manado, Tondano, Indonesia

## Dear Editor,

We read with great enthusiasm the article “Sharing ‘Off-Script’: A Qualitative Analysis of Providers’ Empathic Self-Disclosures During Dignity Therapy” (Mroz et al. 2025). The study offers in-depth insights into how *empathic self-disclosures* (ESDs) are used by healthcare providers in *Dignity Therapy* to improve emotional connection with patients. These findings are particularly relevant in the current development of palliative care, where psychospiritual aspects are increasingly recognized as essential to patient care (Reckson et al. 2023). Understanding ESD is becoming increasingly important with the increasing prevalence of chronic diseases and the need for more personalized therapeutic communication. This correspondence aims to highlight the importance of ESD, examine further implications, and propose optimization strategies in the context of evolving healthcare services.

The central concept raised in this study is the role of ESD in enriching the patient’s *life review* process through the sharing of personal experiences by service providers. In an era of healthcare that is increasingly technology-based and efficient, empathic communication skills are often overlooked. However, this study shows that the humanistic aspects of therapy have a significant impact on the psychosocial well-being of patients, especially those facing terminal illnesses (Liu et al. 2022). Empathy-based approaches such as ESD should receive more attention as person-centered care evolves. With the increasing need for comprehensive palliative care, understanding and developing the right strategies to utilize ESD can help strengthen the patient–provider relationship and improve patients’ quality of life.

While this research provides valuable insights, there is a gap in understanding when and how ESD can be used effectively. While some ESDs can enhance emotional connection and enrich patient reflection (Liaquat et al. 2025), there is also the potential risk of shifting focus from the patient’s narrative to the provider’s experience (Chang et al. 2022). In addition, the study shows that some service providers tend to perform ESD “off-script,” which can indicate a lack of clear guidelines or training on its use. The consequences of this ambiguity can be positive, in the form of flexibility and authenticity of communication, but it can also be damaging if ESD interferes with the patient’s experience of reviewing their lives. Therefore, further research is needed to explore the ideal limits of the use of ESD in *Dignity Therapy*.

More explicit guidelines must be developed regarding when and how service providers can use these strategies (Crowe et al. 2024). One of the solutions is to integrate empathetic communication training into the curriculum of health professionals, especially in the palliative field. Additionally, developing technology-based tools, such as interactive modules or AI-based simulations, can help service providers practice using ESD in safe scenarios before implementing it in actual practice. Furthermore, a longitudinal research-based approach can provide insights into the long-term impact of ESD on the patient experience. Thus, health policies can be designed to support ESD’s practical and ethical adoption in palliative care.

The implications of this study extend to various aspects of health services, including improving clinical communication skills, developing *patient-centered care* policies, and innovating in training health workers. Further understanding of the use of ESD can also be appliedin various other therapeutic contexts, such as psychological counseling and interactions in primary care (Gao et al. 2024). In addition, with increasing attention to psychosocial aspects of global health, ESD-based approaches can help build more meaningful therapeutic relationships between providers and patients. Therefore, investment in research and implementation of empathic communication strategies must be increased to ensure that healthcare services focus not only on physical medication but also on the overall emotional well-being of patients.
